# Predictors of Surgical Intervention for Pediatric Acute Rhinosinusitis with Periorbital Infection

**DOI:** 10.3390/jcm11133831

**Published:** 2022-07-01

**Authors:** Pei-Wen Wu, Yen-Ling Lin, Yun-Shien Lee, Cheng-Hsun Chiu, Ta-Jen Lee, Chien-Chia Huang

**Affiliations:** 1 Division of Rhinology, Department of Otolaryngology, Chang Gung Memorial Hospital, Taoyuan 333, Taiwan; a9665@cgmh.org.tw (P.-W.W.); entlee@cgmh.org.tw (T.-J.L.); 2 School of Medicine, Chang Gung University, Taoyuan 333, Taiwan; chchiu@cgmh.org.tw; 3 Department of Medical Imaging and Intervention, Chang Gung Memorial Hospital, Chang Gung University, Taoyuan 333, Taiwan; gridling@gmail.com; 4 Genomic Medicine Research Core Laboratory, Chang Gung Memorial Hospital, Taoyuan 333, Taiwan; bojack@mail.mcu.edu.tw; 5 Department of Biotechnology, Ming Chuan University, Taoyuan 333, Taiwan; 6 Division of Pediatric Infectious Diseases, Department of Pediatrics, Chang Gung Memorial Hospital, Chang Gung University, Taoyuan 333, Taiwan; 7 Department of Otolaryngology, Xiamen Chang Gung Hospital, Xiamen 102218, China; 8 Graduate Institute of Clinical Medical Sciences, College of Medicine, Chang Gung University, Taoyuan 333, Taiwan

**Keywords:** pediatric periorbital complication, acute rhinosinusitis, intravenous antibiotic treatment, endoscopic sinus surgery, proptosis

## Abstract

Background: Medical versus surgical management of pediatric periorbital infection secondary to acute bacterial rhinosinusitis (ABRS) can be a dilemma for clinicians. This study aimed to evaluate the prognostic factors related to the need for surgical drainage and to help direct management decisions. Methods: Children admitted for periorbital infection secondary to ABRS between 2001 and 2019 were retrospectively reviewed. Demographics, clinical presentations, laboratory data, comorbidities, and computed tomography results were collected from medical records. Results: A total of 141 pediatric patients were enrolled. Forty-two patients (29.8%) required surgical intervention. Multivariate logistic regression analysis identified that delayed initiation of intravenous antibiotics from the onset of periorbital swelling (odds ratio [OR] = 1.94; *p* < 0.001) and proptosis at initial presentation (OR = 6.63; *p* = 0.008) were significantly associated with the need for surgical intervention. A C-reactive protein value of > 55.73 mg/L and initiation of intravenous antibiotic treatment > 2 days from the onset of periorbital swelling showed the best predictive power for surgery. Conclusions: Pediatric patients with delayed initiation of intravenous antibiotic treatment and initial presentation of proptosis had worse outcomes and required surgical intervention.

## 1. Introduction

The periorbital spread of infection is the most common complication of acute bacterial rhinosinusitis (ABRS) that often arises from upper respiratory infections [1]. Periorbital complications (POC) are seen in approximately 6% of patients with ABRS and are more common in children than adults [2]. Even though they usually do not result in mortality, pediatric POC could have significant morbidity such as vision impairment and are more readily treated with aggressive medical management [3]. However, older children are more likely to require surgical intervention and have intracranial complications, which can be potentially life-threatening [1]. It is widely accepted that managing these complications starts with aggressive intravenous antibiotic therapy. If no improvement is seen after 24 h of intravenous antibiotics, or if the patient’s clinical condition worsens, computerized tomography (CT) scans may be required to identify a possible abscess and perform surgical drainage [1,3]. In 1970, Chandler described the different extents of the spread of infection into the periorbital area based on specific clinical findings, which today are used in conjunction with CT to determine the stage of infection [4]. According to Chandler, orbital complications of rhinosinusitis can be classified into five groups, namely: preseptal cellulitis, orbital cellulitis, subperiosteal abscess (SPA), orbital abscess, and cavernous sinus thrombosis [4]. However, there is no established guideline addressing the timing of choosing medical or surgical management, either according to the different Chandler’s stages or specific clinical characteristics. Although the rate of surgical intervention increases with Chandler’s stages, some authors have concluded that this is a poor predictor clinically because of the relatively low overall surgical rate [5]. Further investigation illuminating the clinical predictors prompting early surgical intervention or conservative medical treatment is mandatory in the era of precision medicine.

With improvements in surgical techniques and equipment used for endoscopic sinus surgery (ESS), more procedures are being performed in children and have been proven to be effective and safe in pediatric patients [6,7]. The rate of surgical intervention for complicated pediatric sinusitis has been increasing in the last two decades, ranging from 15.8% to 19.5% [8,9,10]. Children recover quicker with endoscopic drainage, and the hospital stay is approximately half of that when an external approach is used [11]. However, considering the uncertain impact of surgery on facial growth [12,13], the possible dramatic improvement after intravenous antibiotic therapy in some cases, and poor cooperation with postoperative local nasal treatment in children, avoiding unnecessary surgery is as important today as it has always been [6].

Every decision regarding surgery involves a risk–benefit analysis. The early identification of patients who may benefit from ESS drainage is important to prevent ineffective medical treatment and prolonged hospitalization. In this study, we retrospectively evaluated pediatric patients with POC secondary to ABRS. The aims of the study were to evaluate different clinical characteristics to determine when medical management was appropriate and to identify prognostic factors that may contribute to the need for surgical intervention.

## 2. Materials and Methods

### 2.1. Patients

Pediatric patients, aged 0–18, who were admitted to our hospital between 2001 and 2019 with a diagnosis of POC secondary to ABRS, which was defined by documentation in a radiology report, were identified by manual chart reviews. Exclusion criteria included concomitant benign or malignant sinonasal neoplasms; incomplete medical records or radiology images; immuno-compromised patients; invasive fungal sinusitis; dacryocystitis; infection processes secondary to other causes, such as allergic reactions, insect bites, trauma, or odontogenic infection.

### 2.2. Outcome

The primary study endpoint was a surgical intervention. All patients received intravenous antibiotics as the initial treatment when they arrived at the hospital. Subsequent surgical management—including ESS, orbitotomy, or both—was performed if no improvement was seen after 24 h intravenous antibiotics or if the patient’s clinical condition worsened. The extent of ESS varies according to the spread of the infection, which may include middle meatal antrostomy, anterior ethmoidectomy, or anterior and posterior ethmoidectomy. Clinical manifestation, disease severity according to Chandler’s stages, laboratory data at the initial presentation, and the record of ophthalmic exams were all collected for analysis.

### 2.3. Statistical Analysis

Statistical analysis was performed using MATLAB 2015b (The MathWorks, Natick, MA, USA). Patients with and without surgical intervention were then divided into two groups, and the factors related to the decision for surgical drainage were compared. Univariate analysis of the categorical variables was performed using χ^2^ and Fisher’s exact tests, where appropriate. The Mann–Whitney U test was used for continuous variables. Logistic regression analysis was performed to evaluate the association of multiple variables with the necessity of surgical intervention. For each significant independent variable, odds ratios (OR) and 95% confidence intervals (CI) were calculated. To identify and characterize the sensitivity and specificity of the predictors of surgical intervention, receiver operating characteristic (ROC) curves were analyzed and the area under the ROC curve (AUC) was calculated. Statistical significance was set at *p* < 0.05.

### 2.4. Ethics

This study was approved by the institutional review board of Chang Gung Memorial Hospital (approval number: 202000527B0). All study procedures were performed in accordance with the relevant guidelines and regulations. The requirement for informed consent was waived due to the retrospective nature of the study and anonymity of the data.

## 3. Results

Between 2001 and 2019, 141 pediatric patients treated for ABRS with POC were enrolled. None of the patients enrolled in this study had formal sinus surgery. Seventy-four boys and sixty-seven girls were enrolled and had a mean age of 6.94 ± 4.99 years. Forty-two patients (29.79%) needed surgical intervention. Among them, twenty patients received ESS, nine patients had orbitotomy, and thirteen patients had combined surgery. The mean hospital-staying period was 9.74 ± 7.98 days (Table 1). The most common complication was an SPA (Chandler III) in 55 (39%) patients, followed by preseptal cellulitis (Chandler I, 38%), orbital abscess (Chandler IV, 22%), orbital cellulitis (Chandler II, 16%), and cavernous sinus thrombosis (Chandler V, 0.7%). (Table 2).

**Figure 1:**
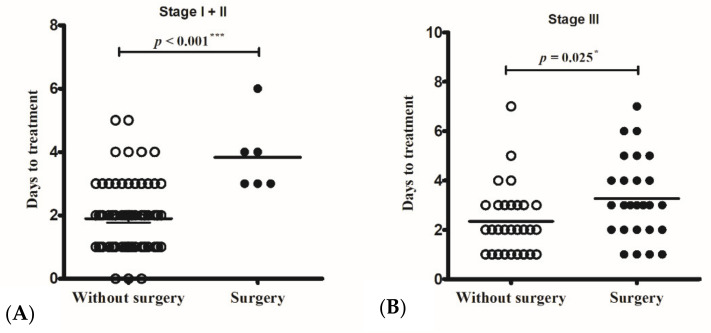
The only operated Chandler I case and the five operated Chandler II cases all started intravenous antibiotic therapy more than three days from symptom onset, whereas non-operated cases started intravenous antibiotic therapy less than two days from symptom onset (*p* < 0.001) (**A**). Non-operated Chandler III cases had intravenous antibiotic therapy (2.34 ± 1.40 days) earlier than those with surgical intervention (3.27 ± 1.66 days) (*p* = 0.025) (**B**). * *p* < 0.05; *** *p* < 0.001.

The operative rates in stage I and II cases were 2% (1/38) and 23% (5/16), respectively. All the operated cases commenced intravenous antibiotic therapy more than three days after the onset of periorbital swelling, whereas the non-operated cases started intravenous antibiotic therapy earlier, 1.91 ± 1.16 days (in stage I) and 1.88 ± 0.86 days (in stage II) from symptom onset (*p* < 0.001) (Figure 1A). The only patient with stage I disease who needed a surgical intervention was a 10-year-old girl, who was sent to the emergency room due to a high-grade fever and periorbital pain for more than three days. Laboratory data on the day of arrival at the hospital showed leukocytosis (19,400/uL) and a high CRP level (78.49 mg/L). Persistent fever and progressed periorbital pain were noted after 24 h of intravenous antibiotic treatment. Endoscopic sinus surgery, including maxillary antrostomy and anterior ethmoidectomy, was performed for drainage.

Twenty-six (47%) cases with an SPA required a surgical intervention. Among these cases, non-operated children received intravenous antibiotic therapy (2.34 ± 1.40 days) earlier than those who received surgical intervention (3.27 ± 1.66 days) (*p* = 0.025) (Figure 1B). All patients with stage IV and V diseases had operations.

Univariate analysis revealed that patients who underwent surgical drainage had longer hospital stays (*p* < 0.001). Higher C-reactive protein (CRP) levels (*p* = 0.001), higher band neutrophils (*p* = 0.004), delayed initiation of intravenous antibiotic treatment (*p* < 0.001), initial clinical presentation with extraocular movement (EOM) limitation (*p* < 0.001), proptosis (*p* < 0.001), and blurred vision (*p* = 0.024) were significantly associated with the need for surgical intervention. There were no significant differences in sex or age between the two groups (Table 3).

In the multivariate logistic regression analysis, after adjustment for age and sex, the delay in the initiation of intravenous antibiotics from the onset of periorbital swelling (OR = 1.94; *p* < 0.001) and proptosis at initial presentation (OR = 6.63; *p* = 0.008) were significantly associated with the need for surgical intervention (Table 4).

**Figure 2:**
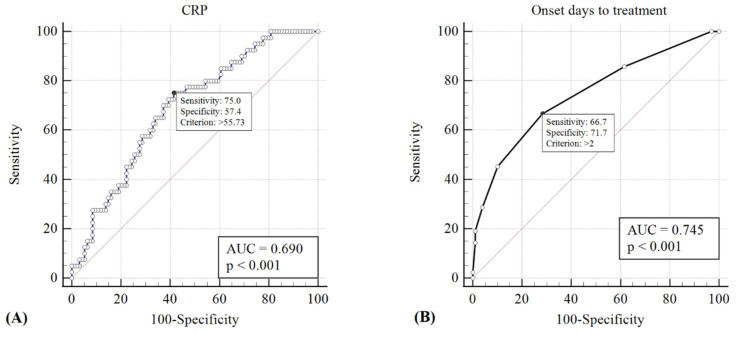
The cut-off points of CRP value >55.73 mg/L (**A**) and initiation of intravenous antibiotics >2 days from onset of periorbital swelling (**B**) showed the best predictive power for the necessity of surgical drainage (sensitivity = 0.75 and 0.67, specificity = 0.57 and 0.72, respectively); the respective AUCs were 0.69 and 0.75 (*p* < 0.001).

ROC curves were generated, and the AUC was calculated to evaluate the sensitivity and specificity of the CRP value and the interval of initiating intravenous antibiotics from symptom onset in detecting patients who require surgical intervention. A CRP value of >55.73 mg/L and initiating intravenous antibiotics >2 days from the onset of periorbital swelling showed the best predictive power for the necessity of surgical drainage (sensitivity = 0.75 and 0.67, specificity = 0.57 and 0.72, respectively); the respective AUCs were 0.69 and 0.75 (*p* < 0.001) (Figure 2). On the other hand, twenty-eight of twenty-nine (96.6%) children who met both criteria of a CRP value <55.73 mg/L and the initiation of intravenous antibiotics <2 days were successfully treated medically, without requiring surgical intervention.

Among the 42 patients that underwent surgical drainage, pathogens according to intraoperative tissue/pus culture results included (listed sequentially): *methicillin-resistant Staphylococcus aureus* (*MRSA*) (33.33%), *Viridans streptococcus* (26.19%), *Streptococcus pneumoniae* (*S. pneumoniae*) (7.14%), *methicillin-susceptible Staphylococcus aureus* (*MSSA*) (4.76%), *Pseudomonas aeruginosa* (2.38%), *Streptococcus mitis* (2.38%), *Group B streptococci* (2.38%), and *Haemophilus influenza* (2.38%). Among these patients, eight (19.05%) showed no growth of specific pathogens. None of the patients developed severe neurological or visual deficits postoperatively.

A total of 136 of 141 patients had blood cultures. Among these patients, 131 (96.32%) showed no growth of specific pathogens. One was positive for S. pneumoniae, one was positive for MSSA, and three were positive for MRSA. The reason for the lack of organisms could be due to early commencement of antibiotics, both in the community and in the hospital.

## 4. Discussion

Medical and surgical management of patients with POC secondary to ABRS is heterogeneous with no clear-cut indications for surgical intervention [8]. In the present study, the operative rates in cases of preseptal orbital cellulitis (2%) and orbital cellulitis (23%) were similar to the typical reported rates of 0–11% and 0–23%, respectively [12]. The one case of an operated Chandler I case and the five cases of operated Chandler II cases commenced intravenous antibiotic therapy more than three days from symptom onset, and all sought medical assistance in the emergency room due to persistent fever. SPA was operated on 47% of the time, within the previously described operative rates for this presentation (14–93%) [14,15,16,17]. Not surprisingly, cases of orbital abscess and cavernous sinus thrombosis all required surgical intervention. We also conducted a search in the Taiwan National Health Insurance Research Database for surgical management in pediatric patients hospitalized due to orbital cellulitis (ICD code: 376.01) secondary to ABRS (ICD codes: 461.0–461.9). Between 2004 and 2013, only 6.25% of children admitted to the hospital for treatment required surgical drainage for orbital infection. It is reasonable that the surgical rate was much lower than that in our study because the data were retrieved from all healthcare units nationwide. The Taiwanese healthcare system is characterized by good accessibility, comprehensive population coverage, and short wait times [18].

In our study, all patients had intravenous antibiotic treatment when they arrived at the hospital. The reason for the delayed commencement of antibiotics was due to delayed presentation to the hospital. We can assume that early hospitalization for intravenous antibiotic treatment could lower the need for surgical drainage. In our ROC analysis, a CRP cut-off value of >55.73 mg/L and initiation of intravenous antibiotics >2 days from the onset of periorbital swelling showed the best predictive power for surgery. The criteria of CRP value less than 55.73 mg/L and initiation of intravenous antibiotics less than 2 days were both met by 96.6% (28/29) of children, who were then successfully treated medically. In conclusion, the early initiation of intravenous antibiotic therapy in pediatric patients with ABRS presenting with periorbital swelling is important to prevent further spread of the infection and avoid surgical intervention.

Proptosis, also known as exophthalmos, is a clinical finding in a wide variety of ocular conditions that can be vision- or life-threatening. Orbital infection secondary to ABRS is the most common etiology in pediatric proptosis [19]. Several studies on the management of orbital cellulitis or SPA all recommend surgical drainage in cases presenting with proptosis [20,21,22,23]. In the present study, univariate analysis revealed that initial clinical presentation with EOM limitation, proptosis, and blurred vision was significantly associated with the need for surgical intervention. Multivariate analysis further identified proptosis as the only independent variable contributing to the increased need for surgical drainage. Proptosis implies infection involving the orbital contents posterior to the orbital septum. The inflammatory process can either directly compress the optic nerve or indirectly decrease the blood supply to the optic nerve, placing the individual at risk of permanent vision loss if not addressed in an expeditious fashion [19]. Our evidence emphasizes the importance of careful and thorough ophthalmic examination in patients presenting with proptosis at the first encounter, despite the early or late stage of the periorbital complication. Once visual acuity deteriorates, surgical intervention for decompression and drainage should be performed immediately. The technical difficulty of assessing visual acuity and color vision in an unwell child has prevented us from recording all possible ophthalmic findings at the initial presentation despite being reviewed by an ophthalmologist. However, following treatment, none of the patients had any visual or cranial nerve impairment. We recommend accurate recording of visual findings with the expertise of a pediatric ophthalmologist where feasible.

In our study, only 7.14% (3/42) of intraoperative cultures contained S. pneumoniae. All three patients, born in 1986, 2001 and 2003, were diagnosed with complicated sinusitis and underwent surgical intervention before 2010. None of them had received the pneumococcal vaccination since the pneumococcal conjugate vaccine (PCV) first became available in Taiwan in late 2005 [24]. This was consistent with published evidence, which revealed a decrease in S. pneumoniae as the leading pathogen in complicated sinusitis in the post-PCV era [25]. However, similar to other invasive pneumococcal diseases, shifting epidemiology and serotype distribution may occur according to different vaccination programs, and continued surveillance is necessary. It is worth noting that one-third of the intraoperative cultures contained MRSA, the most common pathogen in our study population. McCoul et al. reviewed the prevalence of MRSA in non-hospitalized ABRS from 2006 to 2012 and revealed an increasing trend, from 0% to 15.9% [26]. One case series of adult patients with periorbital cellulitis in Taiwan found that MRSA was the most common isolate, and the increasing trend of MRSA was compatible with the reducing efficacy of methicillin against periorbital infection [27]. Although high-dose amoxicillin-clavulanate (90 mg/kg/day every 12 h) is the first choice for complicated sinusitis according to the clinical practice guideline published by the American Academy of Pediatrics in 2013 [1], this evidence emphasizes treating POC secondary to ABRS with broad-spectrum antibiotics with coverage for Streptococcus species and Staphylococcus species, including MRSA. Multidisciplinary teamwork between microbiologists, pediatricians, and otolaryngologists is mandatory in managing this condition.

There was no correlation between a history of asthma/allergic rhinitis/chronic sinusitis and the need for surgical intervention in the present study. Although several conditions are associated with or predisposing to sinusitis, including rhinitis (allergic and non-allergic), viral upper respiratory infection, asthma, and anatomic obstruction [28], none of them were proven to be related to the severity of sinusitis. The atopy of individuals could not predict the spread of the infection, and future studies on acute inflammatory cytokines are needed to clarify the prognosis of complicated sinusitis.

Mahalingam’s study revealed that there was also an association between the early use of intra-nasal decongestants and steroids, resulting in a reduced requirement for surgical intervention [29]. However, the small number of cases, the lack of demographic data comparison, and significant selection bias in the study should be considered. Our institute did not routinely use intranasal decongestants and steroids in this scenario (2001–2019); thus, future studies with prospective randomized designs would be necessary to address the issue of concerning medications in these patients.

This study had several limitations that warrant consideration. First, it was a retrospective study; thus, missing data, the inclusion of different pediatricians and surgeons, and the lack of clinical history were unavoidable complicating factors. Second, information regarding clinical microbiology was obtained from intraoperative cultures. Preoperative antibiotic treatment can lead to false-negative results in microbial analysis. Additionally, the pathogens in the non-operated cases (most patients in this study) were not available. Further investigations regarding the impact of microbes on the need for surgical intervention are required. Finally, this was a retrospective case series with a small sample size. Thus, a large-scale prospective study is required to obtain more information.

## 5. Conclusions

Our study analyzed the prognostic factors related to surgical intervention in 141 pediatric patients with POC secondary to ABRS. We found that patients with delayed initiation of intravenous antibiotic treatment and an initial presentation of proptosis had worse outcomes and required surgical decompression/drainage. However, all patients with Chandler stage I/II/III diseases who had prompt intravenous antibiotic treatment (less than 2 days from the onset of periorbital swelling/pain) could recover without sequelae and without the need for surgery. Finally, the possibility of surgical intervention and poor outcome should be considered by clinicians in patients presenting with proptosis and high CRP; this should be carefully managed with a multidisciplinary approach and should be explained to patients and family members where possible.

## Figures and Tables

**Table 1:** Demographic and descriptive data of entire patient cohort.

Age, years (±SD)	6.94 (± 4.99)
Gender	
Male, N (%)	74 (52.48)
Female, N (%)	67 (47.52)
Hospital-staying period, days (±SD)	9.74 (±7.98)
Symptoms onset to intravenous antibiotics, days (±SD)	2.50 (±1.64)
Surgical intervention, N (%)	42 (29.79)

N = number; SD = standard deviation.

**Table 2:** Extent of disease spread.

Extent of Disease	N (%)	Surgical Rate, N (%)
Chandler’s classification		
Stage I	54 (38)	1 (2)
Stage II	22 (16)	5 (23)
Stage III	55 (39)	26 (47)
Stage IV	9 (22)	9 (100)
Stage V	1 (0.7)	1 (100)

N = number.

**Table 3:** Factors associated with surgical intervention.

Patient Variables	Medical Therapy (N = 99)	Surgical Therapy (N = 42)	*p*-Value (Univariable)
Age, years	6.73 ± 4.90	7.45 ± 5.21	0.435
Hospital-staying period, days	7.65 ± 4.46	14.69 ± 11.59	<0.001 ***
Symptoms onset to parenteral antibiotic, days	2.03 ± 1.20	3.62 ± 1.99	<0.001 ***
Male gender	54 (55%)	20 (48%)	0.467
Symptoms at presentation			
Underlying diseases			
Asthma	3 (3%)	2 (5%)	0.634
Allergic rhinitis	15 (15%)	10 (24%)	0.234
Chronic sinusitis	4 (4%)	3 (7%)	0.425
Fever	72 (72%)	36 (86%)	0.128
Purulent rhinorrhea	65 (66%)	34 (81%)	0.074
Headache	13 (13%)	10 (24%)	0.137
EOM limitation	19 (19%)	25 (60%)	<0.001 ***
Proptosis	32 (32%)	36 (86%)	<0.001 ***
Periorbital pain/swelling	99 (100%)	42 (100%)	1.000
Blurred vision	2 (2%)	5 (12%)	0.024 *
Conscious change	0 (0%)	1 (2%)	0.298
CNS involvement	0 (0%)	4 (%)	0.007 **
Laboratory data at presentation			
WBC, per μL	14,387 ± 5819	15,767 ± 4339	0.170
Segment, %	66.75 ± 17.84	71.89 ± 15.29	0.106
Band form, %	0.28 ± 0.76	1.85 ± 5.17	0.004 **
CRP, mg/L	63.30 ± 57.01	101.14 ± 67.79	0.001 **

N = number; EOM = extraocular movement; CNS = central nervous system; WBC = white blood cell; CRP = C-reactive protein. * *p* < 0.05; ** *p* < 0.01; *** *p* < 0.001

**Table 4:** Multivariate logistic regression analysis of the clinical characteristics related to surgical intervention.

Variable	OR	95% CI	β	SE (β)	*p* Value
Age (years)	0.95	0.852–1.064	−0.048	0.056	0.386
Symptoms onset to intravenous antibiotics (days)	1.94	1.339–2.795	0.660	0.187	<0.001 ***
Gender (male)	0.87	0.325–2.356	−0.133	0.505	0.791
EOM limitation	1.10	0.312–3.906	0.099	0.644	0.877
Proptosis	6.63	1.634–26.909	1.891	0.714	0.008 **
Blurred vision	2.41	0.325–17.914	0.880	1.022	0.389
Band neutrophil	1.34	0.845–2.138	0.295	0.236	0.212
CRP	1.00	0.996–1.012	0.004	0.004	0.283

OR = Odds ratio; CI = confidence interval; EOM = extraocular movement; CRP = C-reactive protein. ** *p* < 0.01; *** *p* < 0.001.

## Data Availability

All data described in the study have been presented in the manuscript. The datasets analyzed are available from the corresponding author on reasonable request.
